# Psychometric Validation of the Community Antimicrobial Use Scale (CAMUS) in Primary Healthcare and the Implications for Future Use

**DOI:** 10.3390/antibiotics15010107

**Published:** 2026-01-21

**Authors:** Nishana Ramdas, Natalie Schellack, Corrie Uys, Brian Godman, Stephen M. Campbell, Johanna C. Meyer

**Affiliations:** 1 Department of Public Health Pharmacy and Management, School of Pharmacy, Sefako Makgatho Health Sciences University, Pretoria 0208, South Africa; stephen.campbell@smu.ac.za (S.M.C.); hannelie.meyer@smu.ac.za (J.C.M.); 2 Department of Pharmacology, Faculty of Health Sciences, University of Pretoria, Pretoria 0007, South Africa; natalie.schellack@up.ac.za; 3 Applied Microbial and Health Biotechnology Institute (AMHBI), Cape Peninsula University of Technology, Bellville, Cape Town 7535, South Africa; uysc@cput.ac.za; 4 Strathclyde Institute of Pharmacy and Biomedical Sciences, University of Strathclyde, Glasgow G4 0RE, UK; 5 Antibiotic Policy Group, Institute for Infection and Immunity, City St. George’s University of London, Cranmer Terrace, London SW17 0RE, UK; 6 School of Health Sciences, University of Manchester, Manchester M13 9PL, UK; 7 South African Vaccination and Immunisation Centre, Sefako Makgatho Health Sciences University, Molotlegi Street, Garankuwa, Pretoria 0208, South Africa

**Keywords:** antimicrobial resistance, knowledge, attitudes, motivations, expectations, primary healthcare, patients, community, antibiotics, South Africa

## Abstract

**Background/Objectives:** Patient-level factors strongly influence antimicrobial resistance (AMR) through the pressure applied to healthcare professionals to prescribe antibiotics even for self-limiting viral infections, enhanced by knowledge and attitude concerns. This includes Africa, with high levels of AMR. However, validated measurement tools for African primary healthcare (PHC) are scarce. This study evaluated the reliability, structural validity, and interpretability of the Community Antimicrobial Use Scale (CAMUS) in South Africa. **Methods:** A cross-sectional survey was conducted with 1283 adults across 25 diverse public PHC facilities across two provinces. The 30-item theory-based tool underwent exploratory and confirmatory factor analysis (EFA/CFA), reliability, and validity testing. **Results:** EFA identified a coherent five-factor structure: (F1) Understanding antibiotics; (F2) Social and behavioural norms; (F3) Non-prescribed use; (F4) Understanding of AMR; and (F5) Attitudes. Internal consistency was strongest for knowledge and misuse domains (alpha approximation 0.80). Test–retest reliability was good-to-excellent (ICC: 0.72–0.89). CFA confirmed acceptable composite reliability (CR ≥ 0.63). Although average variance extracted (AVE) was low for broader behavioural constructs, indicating conceptual breadth, it was high for AMR knowledge (0.737). Construct validity was supported by positive correlations with health literacy (r = 0.48) and appropriate use intentions (r = 0.42). Measurement error metrics (SEM = 1.59; SDC = 4.40) indicated good precision for group-level comparisons. **Conclusions:** CAMUS demonstrated a theoretically grounded structure with robust performance in knowledge and misuse domains. While social and attitudinal domains require refinement, we believe the tool is psychometrically suitable for group-level antimicrobial use surveillance and programme evaluation in South African PHC settings and wider to help with targeting future educational programmes among patients.

## 1. Introduction

Antimicrobial resistance (AMR) represents a major and growing threat to global health security, potentially becoming the next pandemic [1,2,3]. This is especially the case among low-and middle-income countries (LMICs), including African countries, where infectious disease rates remain high and access to appropriate care and antibiotic stewardship is limited [4,5,6,7]. The diminishing efficacy of existing antimicrobial agents compromises the ability to treat common infections effectively, leading to increased morbidity, mortality, and healthcare costs [4,5,8,9,10,11,12].

AMR is driven by a number of factors especially among LMICs. These include high infectious disease prevalence rates, limited diagnostic capacity, suboptimal antibiotic prescribing and dispensing practices, and challenges in regulating access to medicines [2,5,13,14,15]. A primary driver of AMR in LMICs is the inappropriate use of antimicrobials especially in ambulatory care [8,13,16,17,18,19]. Primary healthcare (PHC) facilities are a central point of care for many patients in LMICs seeking treatment from physicians or nurses [8,16,20,21]. However, care-seeking pathways are often pluralistic, and patients, particularly for self-limiting infectious conditions in LMICs, may first consult community pharmacists or informal providers before attending PHC services as there can be challenges with access to PHC facilities [14,20,21,22,23].

PHC facilities in LMICs typically face high patient workloads, compounded by physician shortages, particularly in rural areas [5,8,22]. Alongside this, there can be variable prescribing oversight and strong patient expectations for antibiotics even for self-limiting conditions [2,8,13,14,24]. In South Africa, AMR is recognised as an increasing national priority with mortality directly attributed to resistant infections, now surpassing mortality rates from several other major causes [25,26]. This highlights the urgent need for robust, context-specific interventions in South Africa, especially given ongoing concerns with the current lower priority given by the Ministry of Health to the prevention of, and reduction in, AMR in the country [25,26,27,28]. 

Globally, up to 95% of human antibiotic consumption in LMICs occurs in primary care [19]. Consequently, primary care is a key focus for any national activities to improve future prescribing and reduce AMR [5,8,18,29,30]. In South Africa, PHC facilities typically serve as the first point of contact for most of the population with both infectious and non-infectious diseases [20,21]. There is currently limited if any informal providers in South Africa unlike other LMICs, which includes other African countries [23,31,32,33]. However, as detailed in Supplementary Table S1, published studies, coupled with our own preliminary audit of the study sites, have consistently documented high rates of inappropriate antibiotic prescribing among PHC facilities in South Africa, often for self-limiting viral conditions where they offer no clinical benefit [21,34,35,36,37,38,39,40]. This clinical pattern, where prescribing frequently deviates from guidelines, suggests that non-clinical factors, such as patient pressure and expectations, play a substantial role. These findings directly informed the development of the Community Antimicrobial Use Scale (CAMUS) items, specifically those investigating patient entitlement and the social norms that drive demand for antibiotics.

Patient-level factors do exert significant influence on antibiotic utilisation patterns especially in LMICs [8]. Patients’ knowledge (or lack thereof) about antibiotics, their attitudes towards illness and treatment, personal motivations, expectations regarding consultations, and subsequent health behaviours, all shape demand for, and use of, antibiotics [8,24,41]. Published studies among LMICs, including those from South Africa, indicate that patients frequently pressure clinicians to prescribe antibiotics, enhanced by a misunderstanding of appropriate indications for antibiotics, e.g., believing antibiotics can treat coughs and colds (Table 1 and Supplementary Table S1) [8,41,42].

Despite the widely acknowledged importance of patient perspectives, antimicrobial stewardship (AMS) initiatives in South Africa, alongside many other LMICs, have traditionally focused on hospital settings rather than community settings [5,43,44,45]. However, we are beginning to see more antibiotic stewardship programmes (ASPs) being undertaken among prescribers across LMICs to improve future antibiotic use [8,46]. Having said this, potential AMS interventions targeting patients or the community often lack an evidence base regarding the specific behavioural drivers prevalent in local contexts [8,41]. A major impediment has been the absence of validated, reliable, and culturally appropriate measurement tools designed to capture the multifaceted nature of patient knowledge, attitudes, motivations, and expectations related to antibiotic use, particularly within African healthcare systems [41].

While numerous studies have assessed these factors, they overwhelmingly rely on descriptive knowledge, attitudes, and practices (KAP) surveys [8,15]. As our previously published scoping review confirmed, these existing instruments generally lack a foundation in behavioural theory and have not undergone rigorous psychometric validation [41]. Consequently, the critical gap is not a lack of surveys, but a lack of validated, theory-driven tools. Unlike traditional KAP surveys, which typically offer descriptive snapshots of isolated variables, CAMUS aims to measure the underlying latent constructs, including social pressure and entitlement, that drive behaviour. By moving beyond simple frequency reporting to psychometric structural analysis, CAMUS should offer a reproducible tool to identify the specific behavioural levers requiring intervention [8,41].

Overall, understanding patients’ perspectives is fundamental to designing interventions in South Africa and across LMICs that resonate with community members and effectively modify behaviour. To address this critical need, we conducted a multi-phase research project aimed at developing and validating the CAMUS. The phases, methods, and key findings are summarised in Table 2.

Briefly, the scoping review synthesised global evidence on patient-level drivers of antimicrobial use and highlighted major gaps in theory use and psychometric validation of existing instruments. These findings informed item generation and tool development, which were also guided by the Theory of Planned Behaviour (TPB), the Health Belief Model (HBM) and Social Cognitive Theory (SCT) [47,48]. A pilot study in PHC settings in South Africa subsequently tested the CAMUS for its feasibility, clarity and acceptability, and identified substantial baseline misconceptions and areas for item refinement [49].

While the pilot study established feasibility and content validity, it lacked the statistical power to validate the instrument’s structure. Consequently, the present study was designed as a large-scale psychometric validation. The primary objective was to rigorously evaluate the reliability, structural validity and interpretability of the CAMUS in a large, diverse sample of South African PHC patients. As a result, we sought to establish its suitability as a measure for research and programme evaluation. We chose PHC facilities for this initial study as there had been variable findings regarding the purchasing of antibiotics without a prescription in South Africa. A number of studies at the time of the development of CAMUS suggested little or no purchasing of antibiotics without a prescription [50,51]. However, others demonstrated some purchasing of antibiotics without a prescription, although typically not for self-limiting viral conditions such as upper respiratory tract infections [52].

**Table 2:** Overview of the multi-phase CAMUS development and validation study.

Step	Phase/Study Component	Aim	Methods/Key Activities	Main Findings and Outputs	References
**1**	Scoping review of patient-level drivers of antimicrobial use	To identify knowledge, attitudes, motivations, expectations, social and behavioural norms and structural factors influencing AMU, and to map existing measurement tools	Systematic scoping review of studies on community-level AMU and AMR, with extraction of behavioural constructs and survey instruments	Widespread misconceptions about antibiotic effectiveness (e.g., for viral infections), strong patient expectations for antibiotics, and important roles for social and behavioural norms and system pressures. Existing instruments were largely descriptive KAP tools with limited theoretical grounding and almost no psychometric validation.	[8,41]
**2**	Tool development (CAMUS item generation and refinement)	To develop a theory-informed instrument capturing key behavioural domains relevant to AMU in PHC	Item generation based on scoping review findings; expert review; mapping of items to constructs from the Theory of Planned Behaviour (TBP), Health Belief Model (HBM) and Social Cognitive Theory (SCT); iterative refinement for content validity	Draft 30-item CAMUS instrument covering knowledge, attitudes, motivations, expectations and behavioural practices, grounded in TPB, HBM and SCT.	[48,53,54]
**3**	Pilot study of CAMUS in PHC patients	To assess feasibility, clarity and acceptability of the draft 30-item CAMUS in routine PHC settings	Administration of CAMUS to 30 adult PHC attendees; timing of completion; cognitive interviewing; item-level feedback	CAMUS was feasible in clinic workflows (mean completion time ≈10 min) and acceptable to patients. Pilot data showed marked misconceptions (e.g., 93.3% believed antibiotics treat colds/coughs; 43.3% recognised that overuse causes resistance) and led to refinement of 28 items for clarity.	[41]
**4**	Large-scale psychometric validation (current study)	To evaluate reliability, structural validity and interpretability of CAMUS in a large PHC population	Cross-sectional survey of 1283 adult PHC attendees in Gauteng and Limpopo; Exploratory Factor Analysis; Confirmatory Factor Analysis; internal consistency; test–retest reliability; construct and convergent validity; measurement error (SEM/SDC)	Five-factor structure reflecting knowledge, social and behavioural norms, non-prescribed use, AMR understanding and expectations/beliefs; acceptable–good reliability for most domains; good test–retest stability; construct validity via associations with health literacy and intentions; SEM/SDC supporting use at group level.	Current study

NB: AMR, antimicrobial resistance; AMU, antimicrobial use; CAMUS, Community Anti-Microbial Use Scale; KAP, knowledge, attitudes, and practices; PHC, primary healthcare; SDC, smallest detectable change; and SEM, standard error of measurement.

## 2. Results

### 2.1. Participant Characteristics

A total of 1283 participants were enrolled and completed the CAMUS questionnaire. The demographic profile of the sample is summarised in Table 3.

The majority of participants were female (59.3%), and the largest age groups were 20–29 years (34.3%) and 30–39 years (24.2%). Participants primarily identified themselves as African (89.4%) and residing in townships (46.2%) or rural areas (30.2%). Educational attainment varied, with 46.0% having completed high school. In total, 42.1% of the sample was employed, while 35.5% reported being unemployed.

### 2.2. Phase 1: Exploratory Factor Analysis (EFA) and Test–Retest Reliability

The dataset was suitable for factor analysis (KMO = 0.867; Bartlett’s Test of Sphericity χ^2^(435) = 10,751.464; *p* < 0.001). EFA was conducted on the 30 CAMUS items following categorical principal components transformation. Principal axis factoring with Promax rotation identified a five-factor solution that aligned well with the theoretical domains specified in advance. Box 1 contains the final structure of CAMUS.

Box 1 **Final factor structure of the CAMUS.**

- F1. Understanding of antibiotics
  General knowledge regarding indications, effectiveness, and side effects of antibiotics.
- F2. Social and behavioural norms related to antibiotic use
  Perceived family, community, and social influences shaping antibiotic use behaviours.
- F3. Non-prescribed use
  Informal practices including sharing, storing, and using antibiotics without prescription.
- F4. Understanding of AMR
  Knowledge of resistance mechanisms and consequences of inappropriate antibiotic use.
- F5. Attitudes and beliefs toward antibiotics
  Expectations, entitlement beliefs, and attitudes influencing demand for antibiotics.

This structure explained a substantial proportion of the variance and showed clear conceptual coherence: F1 captured core knowledge about indications and mechanisms; F2 grouped items on family and community norms and interactions with providers; F3 captured informal or non-prescribed use; F4 measured AMR-specific knowledge; and F5 encompassed expectations and entitlement beliefs. The rotated pattern matrix and corresponding factor loadings for this five-factor solution are presented in Table 4.

Internal consistency for these exploratory factors was also explored:

- F1 Understanding of antibiotics: α = 0.806 (7 items).
- F2 Social and behavioural norms: α = 0.767 (9 items).
- F3 Non-prescribed use: α = 0.801 (6 items).
- F4 Understanding of AMR: α = 0.798 (3 items).
- F5 Attitudes and beliefs: α = 0.542 (3 items).

Consequently, three constructs (F1, F3, F4) showed good internal consistency. F2 showed acceptable to good reliability for a broad social construct, whilst F5 demonstrated marginal reliability in this preliminary phase due to both construct breadth and low item numbers (*n* = 3).

As part of the refinement process, five Likert items (Items 1, 2, 5, 14, and 19) were removed prior to conducting the CFA due to low communalities (<0.30), cross-loadings <0.30 on multiple factors, or conceptual ambiguity (weak primary factor loadings <0.40). Their removal improved the coherence of the factor structure without compromising theoretical coverage.

Test–retest reliability, based on a subsample of 69 participants re-assessed after 5–7 days, was good to excellent across the CAMUS domains (Table 5), with ICCs ranging from 0.72 (Attitudes and beliefs) to 0.89 (Understanding of AMR)**.** Table 4 contains details of the rotated pattern matrix and factor loadings for the five-factor EFA solution.

### 2.3. Phase 2: Confirmatory Factor Analysis (CFA)

**Figure 1:**
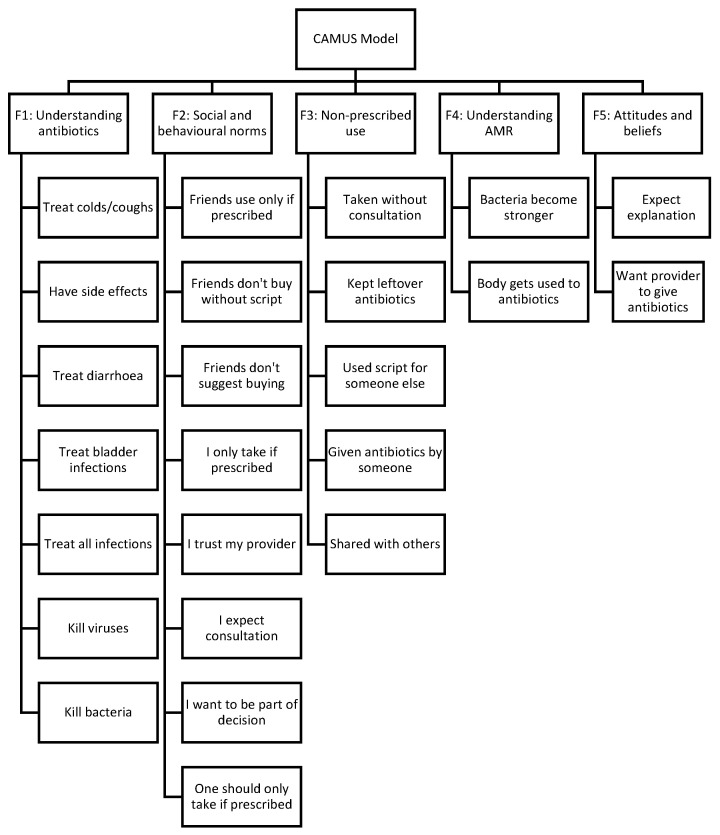
Confirmatory Factor Analysis (CFA) path diagram of the refined 5-factor CAMUS model. NB: The diagram illustrates the relationship between the five latent constructs (F1–F5) and their respective questionnaire items.

The final CFA model structure is illustrated in Figure 1. The model comprises five latent factors: Understanding of antibiotics (F1); Social and behavioural norms (F2); Non-prescribed use (F3); Understanding of AMR (F4); and Attitudes and beliefs (F5).

Table 6 summarises the CFA-based reliability and convergent validity indices. Three constructs (F1, F3, F4) achieved CR ≥ 0.79 with alpha ≥ 0.67, indicating good internal consistency and composite reliability (CR). F2 and F5 had CR in the 0.63–0.67 range and alpha around 0.62–0.73, which is acceptable for early-stage behavioural scales, particularly for shorter subscales.

For broad social/behavioural domains, average variance extracted (AVE) values were below 0.50 for F1, F2, and F3, while F4 (Understanding of AMR) showed excellent convergent validity (AVE = 0.737), and F5 approached the conventional 0.50 threshold (AVE = 0.471). While AVE values for the social and behavioural norm factors (F1, F2, F3) were below the strict threshold of 0.50, the CR for these constructs exceeded 0.60. According to the divergent validity criteria established by Fornell and Larcker (1981) [47], an AVE less than 0.50 is acceptable provided that the CR is higher than 0.60, indicating that the convergent validity of the construct is still adequate.

### 2.4. Construct and Convergent Validity

Construct validity was supported by associations with theoretically related variables. Among the subsample who completed the health literacy test (HELT-LL, *n* = 463), CAMUS factor scores correlated moderately and positively with health literacy (r = 0.48, *p* < 0.001) and with self-reported intentions to use antibiotics appropriately (e.g., finish prescribed courses, avoid use without prescription; r = 0.42, *p* < 0.01). Participants with higher health literacy demonstrated better antibiotic knowledge and more appropriate attitudes and intentions, consistent with expectations.

### 2.5. Interpretability and Measurement Error

Measurement error was calculated for each factor independently to account for the multidimensional nature of the scale. As shown in Table 6, the standard error of measurement (SEM) and smallest detectable change (SDC) varied across domains. The average SEM across all factors was 1.59, and the average SDC was 4.40. Using the criterion that acceptable precision requires SEM ≤ SD/2 [54], factors F1 (Understanding antibiotics), F3 (Non-prescribed use), and F4 (Understanding of AMR) demonstrated acceptable precision. However, F2 (Social and behavioural norms) and F5 (Attitudes and beliefs) exhibited higher measurement error (SEM > SD/2), indicating that scores in these specific social-attitudinal domains are subject to greater random variation and should be interpreted with caution at the individual level.

## 3. Discussion and Next Steps

This study provides a large-scale psychometric evaluation of the CAMUS, a theory-informed instrument developed to measure patient-level drivers of antimicrobial use in South African PHC settings. This builds on the findings from the pilot study, which demonstrated that the CAMUS was feasible and acceptable for use in PHC clinics, with a mean completion time of approximately 10 min [49]. The pilot study also revealed substantial baseline misconceptions regarding antibiotic use, including a high prevalence of incorrect beliefs about antibiotics treating viral infections [49]. Alongside this, the pilot study also identified marginal health literacy as common among PHC attendees and highlighted the influence of patient expectations and social and behavioural norms on antibiotic-seeking behaviour, informing item refinement prior to large-scale validation [49]. Using data from 1283 PHC users, the present study systematically assessed the instrument’s dimensionality, reliability, validity, and interpretability.

### 3.1. Behavioural Structure of Antimicrobial Use

The EFA and subsequent CFA confirmed that antimicrobial use behaviour among PHC patients is multidimensional. The refined model comprises five inter-related but distinct constructs: Understanding of antibiotics; Social and behavioural norms; Non-prescribed use; Understanding of AMR; and Attitudes and beliefs towards antibiotics. This structure is consistent with both the scoping review and the behavioural frameworks underpinning CAMUS. The emergence of distinct factors for ‘understanding antibiotics’ (F1) and ‘social and behavioural norms’ (F2) empirically supports our theoretical integration. This confirms that patients distinguish between the cognitive beliefs derived from the HBM and the social pressures described in SCT and TPB as visualised in our theoretical framework. As a result, reinforces the fact that antibiotic-related behaviour is not reducible to knowledge alone [41,48,55].

The separation of knowledge, social and behavioural norms, misuse practices, AMR understanding, and attitudes/expectations supports a more nuanced view of antibiotic use than traditional KAP surveys. The findings show that patients may simultaneously hold accurate knowledge about some aspects of antibiotics, e.g., their side effects, while maintaining misconceptions about viral infections or engaging in informal sharing and storage. This complexity mirrors reported findings from other LMIC contexts [8] but with greater structural resolution. For example, Nepal et al. (2019) reported that 47.7% of community members in the Rupandehi District of Nepal believed antibiotics help recovery from colds and coughs [56], while a systematic review by Torres et al. (2019) found that misconceptions regarding viral infections in LMICs vary widely [57]. There were similar findings in the reviews of Belachew et al. (2021), Yeika et al. (2021) and Saleem et al. (2025) [8,15,58,59]. Some studies in the review of Torres et al. reported misconception rates as high as 80% [57]. This places the current study’s finding (76.5%) at the upper end of global concern. Unlike the unitary measures often used in these reviews, the CAMUS structurally separates these knowledge deficits from social drivers, offering a more nuanced target for intervention. This emphasises the need for multi-component interventions when looking to improve antibiotic use in primary care.

Despite the statistical distinction between the factors, we acknowledge the conceptual proximity between social and behavioural norms (F2) and attitudes and beliefs (F5), as individual expectations are often shaped by broader societal narratives. However, retaining them as separate domains does appear to be behaviourally critical to guide future ASPs. Social and behavioural norms (F2) capture the extrinsic pressure to conform to group behaviour (e.g., ‘my family expects me to get antibiotics’), whereas attitudes and beliefs (F5) capture the intrinsic sense of entitlement and urgency, e.g., ‘I have a right to demand treatment’. Differentiating between patients acting out of social compliance versus those acting out of personal entitlement leads to future ASPs being based on the most appropriate levers; i.e., utilising community-wide messaging to shift norms versus for instance training clinicians in conflict resolution to manage individual entitlement during consultations.

### 3.2. Reliability and Internal Consistency

Reliability analysis demonstrated that the CAMUS performs best in domains where constructs are more concrete and behaviourally specific. Understanding of antibiotics (F1), non-prescribed use (F3), and understanding of AMR (F4) all showed good internal consistency (α ≈ 0.80) and strong composite reliability. As a result, indicating these domains can be used confidently in future research and programme evaluations.

When evaluated against the conventional threshold of α ≥ 0.70, the social and behavioural norms domain (F2) showed acceptable reliability (α = 0.732; CR = 0.672). However, the shorter attitudes and beliefs construct (F5) demonstrated marginal reliability (α = 0.619; CR = 0.635). It is important to remind readers, including researchers, that reliability coefficients are inherently sensitive to scale length and construct breadth; shorter scales often yield lower alpha values even when the underlying construct is valid. In this context, while the F5 value falls below the 0.70 standard, Hair et al. (2019) [60] note that values between 0.60 and 0.70 are acceptable in exploratory research, particularly for newly developed scales. We attribute this lower value to the sensitivity of Cronbach’s alpha to the number of items, e.g., Factor 5 contains only two items in the final model alongside the broad nature of ‘entitlement beliefs’.

Consequently, we do not claim full psychometric adequacy for this specific subscale in its current form. Instead, these results indicate that F5 requires item expansion. Specifically, adding items related to patient entitlement and expectations, to improve internal consistency in future iterations.

The good to excellent test–retest reliability (ICC 0.72–0.89) provides further evidence that CAMUS domains are stable over short periods, supporting their development and future use for monitoring and evaluating future ASP interventions.

### 3.3. Confirmatory Factor Analysis and Construct Validity

The CFA did not simply replicate the exploratory structure but refined it. Splitting the broad social/behavioural factor into a norms construct and a compact attitudes and beliefs construct improved structural coherence, while retaining alignment with TPB, HBM, and SCT. The resulting five-construct model is theoretically consistent and empirically supported at the factor-level.

We acknowledge that the AVE values for the social and behavioural norms (F2), non-prescribed use (F3), and attitudinal (F5) domains fell below the standard threshold of 0.50 (Table 6). This indicates that the variance captured by these latent constructs is lower than the variance attributable to measurement error. However, the CR for these constructs exceeded 0.60. According to the criterion established by Fornell and Larcker (1981) [47], convergent validity is considered adequate when CR is sufficient, even if AVE falls below 0.50. This suggests that, while the items in these broad behavioural domains are heterogeneous, they share sufficient common variance to be reliable as a composite measure.

Nevertheless, we interpret these domains as developmental rather than fully adequate. The measurement properties observed here, specifically the lower variance in social domains compared to knowledge domains, mirror the findings from other health behaviour instruments validated in African settings, such as HIV adherence scales. This reflects the inherent complexity of capturing multifaceted social drivers relative to fixed knowledge traits. Consequently, these specific subscales should be viewed as preliminary indicators suitable for identifying broad group-level trends, rather than precise diagnostic tools for individuals. Achieving robust convergent validity in these social and attitudinal domains will require future refinement, specifically the generation of more homogenous items to better capture the ‘perceived entitlement’ and ‘social pressure’ constructs.

Construct validity was reinforced through moderate correlations with health literacy and antibiotic-use intentions, showing that CAMUS scores behave as theoretically expected. Participants who understand health information better are also more likely to score higher on knowledge, appropriate attitudes, and intentions regarding antibiotic use.

### 3.4. Interpretability and Intended Use

The absence of floor/ceiling effects, the near-normal distribution of total scores, and the low SEM/SDC values collectively support the interpretability of CAMUS scores. The instrument is sensitive enough to distinguish levels of antibiotic-related knowledge and behaviour across a broad PHC population and to detect meaningful shifts at the group level, for example, before and after public awareness campaigns or stewardship interventions. This psychometric capability distinguishes the CAMUS from descriptive surveillance tools, such as the WHO Multi-Country Public Awareness Survey [61]. While the WHO instrument provides valuable aggregate prevalence data for global benchmarking, it was not designed as a scalar instrument to measure individual-level behavioural change over time. By providing validated score distributions and cut-offs, the CAMUS fills this methodological gap, offering a mechanism to evaluate the specific impact of stewardship interventions in a way that descriptive surveys cannot.

At the same time, measurement error estimates suggest caution when interpreting small changes at the individual level. The CAMUS is best understood as a surveillance and research tool for population-level monitoring and evaluation, rather than as a diagnostic instrument for individual clinical decision-making. This distinction is important and should guide how the tool is deployed in practice in the future to help target specific AMS activities to improve future antibiotic use.

### 3.5. Implications for Policy, Practice, and Patient Engagement

The validated structure and reliability of the CAMUS have several implications as demonstrated in Table 7.

### 3.6. Future Directions and Next Steps

Table 8 summarises the validation of the findings to date and the next steps to take the CAMUS forward.

#### 3.6.1. Instrument Refinement

The immediate next step is a detailed item-level review of the CFA and EFA data. This analysis will form the basis for future instrument development.

This involves identifying and revising or excluding items that showed low factor loadings, high cross-loadings, or contributed to weaker reliability and convergent validity, particularly within the social/behavioural norms (F2) and attitudes and beliefs (F5) domains. For Factor 5, the marginal reliability suggests that the current two items are insufficient to fully capture the construct. Future development will involve generating 3–5 additional items focusing on ‘perceived rights to healthcare’ and ‘expectations of speed of recovery’ to strengthen the homogeneity and reliability of this domain.

The goal is to develop a shorter, more streamlined and psychometrically stronger refined iteration of CAMUS that preserves the well-performing knowledge, AMR and misuse domains whilst seeking to improve measurement precision for social and attitudinal constructs.

#### 3.6.2. Operationalization in Future ASPs

To improve translational relevance, the CAMUS is designed to be operationalized within routine ASPs in three distinct ways:

1. Diagnostic triage: Facility managers can use the scale to identify which specific driver is problematic in their catchment area. For example, a facility scoring low on understanding of antibiotics (F1) would benefit from direct patient education, e.g., posters, and pamphlets, whereas a facility scoring high on social and behavioural norms (F2) would require broader community engagement campaigns to appreciably change societal expectations.
2. Benchmarking: Districts can use the CAMUS to benchmark facilities against one another, identifying ‘hotspots’ of high patient entitlement (F5) that may require additional support for clinicians in conflict resolution.
3. Impact evaluation: As a validated measure with known sensitivity, CAMUS can potentially serve as a pre- and post-intervention assessment tool to quantify the effectiveness of public health campaigns.

#### 3.6.3. Re-Validation

Once a refined version of the instrument is proposed, it must be subjected to a new, full validation study on an independent dataset. This new validation must repeat the entire process, i.e., EFA to identify the new factor structure, followed by a CFA to confirm it. The aim is to produce a final, validated tool that achieves acceptable model fit, strong internal consistency (alpha > 0.7), and good convergent validity (AVE > 0.5).

#### 3.6.4. Future Use in South Africa (Post-Refinement)

The future application of CAMUS following refinement and validation can be summarised across three key areas, as outlined in Table 9:

The 30-item CAMUS represents essential groundwork for measuring community antimicrobial use. These validation findings provide the foundation for a refined, psychometrically promising tool suitable for future surveillance and intervention planning.

### 3.7. Strengths and Limitations

#### 3.7.1. Strengths

This study contributes to the field by offering a psychometrically validated instrument specifically tailored to the resource-limited context of South African primary care. Unlike many existing KAP surveys, the CAMUS is grounded in established behavioural theory (TPB, HBM, SCT), allowing for a structural analysis of the drivers of antibiotic use rather than simple descriptive reporting. The large sample size (*n* = 1283) and the inclusion of diverse demographic groups across two provinces strengthen the stability of the factor structure. Furthermore, the calculation of measurement error metrics (SEM/SDC) provides practical benchmarks for future interventional research.

#### 3.7.2. Limitations

We are aware that several limitations should be noted. While reliability at the factor level was generally acceptable, convergent validity was suboptimal for some behavioural constructs, reflecting conceptual breadth and indicating a need for further item refinement. The cross-sectional design also precluded assessment of predictive validity and responsiveness to change; these measurement properties will require longitudinal and pre–post intervention studies. Reliance on self-reported behaviours introduces the potential for social desirability bias, particularly for socially undesirable practices such as sharing leftover antibiotics or stopping treatment early. In addition, sample sizes within certain demographic subgroups, especially participants with very low education levels, were insufficient to support multi-group confirmatory factor analysis, limiting the assessment of measurement invariance across education categories.

Finally, the administration of the CAMUS exclusively in English represents a significant limitation given the rich socio-linguistic diversity of South Africa and the wider African continent. While English was chosen to ensure standardisation during this initial validation phase, this likely introduced a selection bias favouring participants with higher functional literacy and English proficiency. Consequently, our findings may not fully capture the extent of misconceptions or specific cultural drivers of antibiotic use present in non-English speaking sub-populations. Future research must prioritise the translation, cultural adaptation, and validation of the CAMUS into indigenous languages (e.g., isiZulu, Setswana) to ensure the tool is inclusive and truly representative of the PHC population in South Africa.

## 4. Methods

This study employed a cross-sectional survey design for the psychometric validation of the CAMUS instrument, building on the findings from the pilot study.

### 4.1. The CAMUS Instrument

The development of the CAMUS was grounded in an integrated framework drawing on the TPB, the HBM, and SCT as described in Ramdas et al. (2025) [49]. These theories were selected as complementary lenses to capture the multidimensional nature of antibiotic use. While the TPB maps the path from attitude to intention, it often overlooks specific knowledge gaps. In view of this, we integrated the HBM into our approach to help capture cognitive misconceptions, e.g., ‘antibiotics cure flu’, that drive perceived benefits.

**Figure 2:**
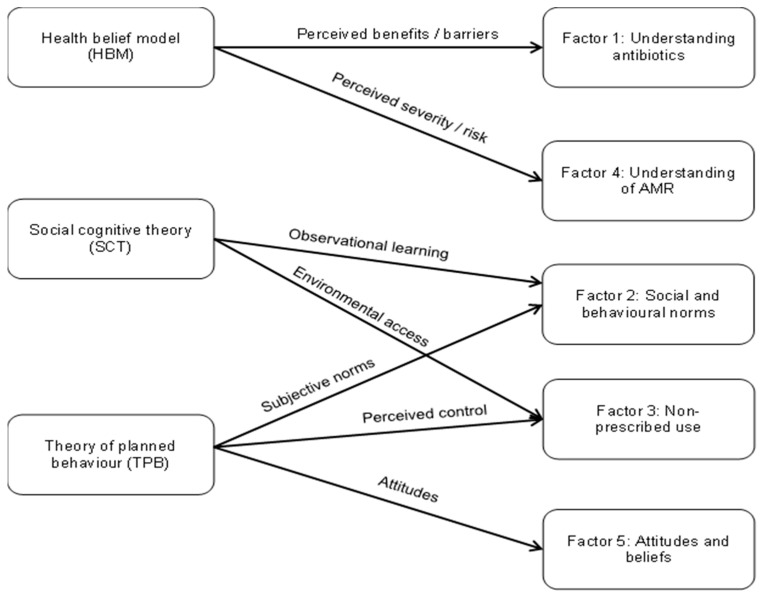
Conceptual framework of the CAMUS instrument.

To account for environmental and social learning prevalent in South African communities, e.g., sharing leftover antibiotics, we incorporated SCT constructs, which map directly to the social and behavioural norms and non-prescribed use domains. Figure 2 illustrates how these theoretical constructs were explicitly mapped to the final CAMUS factors.

The diagram illustrates the integration of theoretical constructs into the final factor structure. Arrows indicate hypothesised directional relationships between theoretical constructs and CAMUS domains. The figure though does not represent a causal model. The HBM informed the cognitive domains regarding risk and antibiotic function (Factors 1 and 4). SCT and the TPB informed the behavioural and social domains, linking observational learning and subjective norms to Factor 2, and environmental access and perceived control to Factor 3. Attitudes (TPB) mapped directly to Factor 5.

In summary, the instrument comprises five core domains designed to capture patient perspectives on AMU (Table 10).

### 4.2. Validation Study Design and Sample

The main validation survey was conducted between August 2024 and September 2025. Participants were adult patients (aged 18 years or older) attending routine (non-urgent) consultations at 25 public PHC facilities. These sites were purposively selected from geographically distinct areas within two provinces (urban, peri-urban, and semi-rural) to build upon the findings from the pilot study, and to capture a diverse patient population representative of the South African PHC setting. The two provinces chosen were Gauteng and Limpopo.

This study focused on PHC facilities within the public health sector, as they are the first point of contact for most South Africans and the primary source of prescribed antibiotics. Whilst we are aware that there are sales of antibiotics without a prescription among community pharmacies in South Africa, the literature on its prevalence in the country is conflicting [50,51,52]. Most studies prior to the development of the CAMUS showed very low rates of purchasing of antibiotics without a prescription in South Africa [50,51]; however, others have shown higher rates [52]. Given these conflicting findings, coupled with the current illegal nature of such sales, we only targeted public sector PHC clinics to robustly measure patient-prescriber interactions and the behavioural drivers (like expectations and motivations) that influence prescribed antibiotic use.

Consecutive sampling was employed in the waiting areas of PHC clinics. Patients were excluded if they were seeking emergency care. This is because this study focused on routine, non-urgent PHC consultations rather than acute urgent conditions, which would typically be managed at a hospital outpatient department. Patients were also excluded if they were too unwell to participate, unwilling to provide informed consent, or unable to communicate in English. English was used as the language of administration for this initial validation study to ensure standardisation and allow for potential international comparison with English recognised as the universal scientific language. This is a recognised approach, given the linguistic diversity in South Africa, and validated translations are a recommended next step.

A total of 1283 participants meeting the inclusion criteria provided informed consent and were enrolled. This sample size comfortably exceeds the standard recommendation of at least 10 participants per scale item, providing sufficient statistical power for robust factor analysis.

### 4.3. Data Collection Procedures

Trained fieldworkers administered the CAMUS questionnaire in English via face-to-face interviews lasting approximately 10–15 min. Responses were captured directly onto electronic tablets using Google Forms. Demographic information was also collected.

For the assessment of test–retest reliability, a randomly selected subsample of 69 participants from one site was asked to complete the CAMUS questionnaire a second time 5 to 7 days after the initial administration.

Additionally, a subset of participants (*n* = 463) completed the Health Literacy Test for Limited Literacy Populations (HELT-LL), previously validated in South Africa, to provide an external criterion for assessing construct validity.

### 4.4. Statistical Analysis

Data were analysed using IBM SPSS Statistics for Windows, Version 30.0 (IBM Corp., Armonk, NY, USA). Table 11 summarises the statistical analyses undertaken to evaluate the psychometric properties of CAMUS. Detailed formulas and citations for the derivation of measurement error metrics (SEM and SDC) are provided in Supplementary Box S1.

### 4.5. Ethical Considerations

The study protocol was approved by the Sefako Makgatho University Research Ethics Committee (SMUREC/P/220/2023:PG). Permissions were obtained from the relevant Provincial Departments of Health. Participation was entirely voluntary. Fieldworkers explained the study purpose, procedures, confidentiality measures, and the right to withdraw at any time without consequence. Written informed consent (or witnessed verbal consent for participants unable to write) was obtained from every participant before enrolment (Supplementary Table S2). Data were anonymized using unique study identifiers, and electronic data were stored securely on password-protected servers accessible only to the core research team.

**Table 11:** Statistical analyses conducted for psychometric validation of CAMUS.

Psychometric Property	Analysis Performed	Statistical Method/Criteria	Purpose
Descriptive statistics	Frequencies, percentages, means, standard deviations	Distributional summaries of items and domain scores	To characterise the sample and response patterns
Internal consistency reliability	Cronbach’s alpha	α ≥ 0.70 considered acceptable	To assess internal consistency of CAMUS domains
Test–retest reliability	ICC	Two-way mixed-effects model, absolute agreement; interpreted per Koo and Li [62]	To assess temporal stability over 5–7 days (*n* = 69)
Sampling adequacy	KMO measure	KMO ≥ 0.60	To assess suitability of data for factor analysis
Sphericity	Bartlett’s Test of Sphericity	*p* < 0.05 indicates factorability	To confirm sufficient inter-item correlations
Data transformation	CatPCA	Transformation of nominal and ordinal variables	To accommodate mixed item response formats
Exploratory factor analysis	Principal axis factoring with Promax rotation	Eigenvalues > 1.0 and theoretical interpretability	To explore underlying factor structure of CAMUS
Confirmatory factor analysis	Reliability and validity diagnostics	CR, AVE, Cronbach’s alpha	To assess convergence and internal consistency of the refined five-factor model
Subgroup analysis	Multi-group CFA	Not performed; subgroup *n* < 100	To document rationale for not testing measurement invariance
Construct validity	Correlation analysis	Pearson’s correlation coefficients	To assess associations with health literacy (HELT-LL) and behavioural intentions

NB: AVE, average variance extracted; CAMUS, Community Antimicrobial Use Scale; CatPCA, categorical principal component analysis; CR, composite reliability; HELT-LL, Health Literacy Test for Limited Literacy populations; ICC, intraclass correlation coefficient; and KMO, Kaiser–Meyer–Olkin.

## 5. Conclusions

This study presents a rigorous, large-scale psychometric evaluation of the CAMUS among PHC patients in South Africa. The instrument demonstrates a coherent five-factor structure grounded in behavioural theories, good internal consistency for core knowledge and misuse domains, acceptable reliability for social and attitudinal domains, good to excellent test–retest stability, and supportive evidence of construct validity via associations with health literacy and antibiotic-use intentions.

Measurement error and distributional properties indicate that CAMUS is well suited to group-level assessment, surveillance, and evaluation of patient-level drivers of antibiotic use, while small individual-level changes should be interpreted with caution. The weaker convergent validity and lower reliability in some behavioural domains identify clear targets for item refinement and future scale development.

CAMUS emerges as a psychometrically promising, contextually relevant tool that can support more behaviourally informed ASP activities in South African PHC settings and, with further adaptation, in other LMIC settings. Future work should focus on item refinement in social and attitudinal domains, translation and cultural validation in major local languages, testing in diverse geographical settings, and longitudinal use to assess responsiveness to stewardship interventions.

## Figures and Tables

**Table 1:** Key patient-level (demand-side) issues influencing antimicrobial resistance in low-and middle-income countries.

Key Issue	Evidence from Low- and Middle-Income Countries Including South Africa
Patient pressure	Clinicians and nurses in South Africa and other low-and middle-income countries frequently cite patient pressure as a primary reason for prescribing antibiotics against their better judgement [8,35,42].
Poor knowledge	Widespread misconceptions exist among patients, which include their beliefs that antibiotics are effective for viral infections including common colds and influenza [8,21,24].
Concerned or risky behaviours	Self-medication with leftover antibiotics, sharing antibiotics with family members, and failing to complete a prescribed course of antibiotics are common [41].

**Table 3:** Participant demographics (*N* = 1283).

Characteristic	Category	Frequency (*n*)	Percentage (%)
Age Group	18–29	481	37.5
	30–39	309	24.1
	40–49	195	15.2
	50–59	148	11.5
	60–69	101	7.9
	70–89	49	3.8
	Total	1283	100
Sex	Male	505	39.4
	Female	771	60.1
	Prefer not to disclose	7	0.5
	Total	1283	100
Ethnicity	African	1153	89.9
	Coloured	31	2.4
	Indian/Asian	23	1.8
	White	76	5.9
	Total	1283	100
Education	No education	33	2.6
	Primary school completed	198	15.4
	High school completed	589	45.9
	College or FTE qualification	233	18.2
	University diploma	98	7.6
	University undergraduate degree	87	6.8
	University postgraduate degree	30	2.3
	Other	15	1.2
	Total	1283	100
Employment	Employed	532	41.5
	Self-employed	151	11.8
	Retired/pensioner	142	11.1
	Unemployed	458	35.7
	Total	1283	100
Residence	Formal urban areas	299	23.3
	Townships	596	46.5
	Rural Areas	388	30.2
	Total	1283	100
Province	Gauteng	876	68.3
	Limpopo	401	31.3
	Other Provinces	6	0.5
	Total	1283	100

**Table 4:** Rotated pattern matrix and factor loadings for the five-factor EFA solution.

Domain	Item	Factor Loading
F1: Understanding of antibiotics	Used to treat colds and coughs	0.738
	Have side effects	0.681
	Used to treat diarrhoea	0.688
	Used to treat bladder infections	0.644
	Can treat all infections	0.711
	Kill viruses	0.641
	Kill bacteria	0.653
F2: Social and behavioural norms	Friends/family use only if prescribed	0.631
	Friends/family do not buy without prescription	0.559
	Friends/family do not suggest buying	0.59
	I only take antibiotics if prescribed	0.612
	I trust my healthcare provider	0.581
	I expect explanation from provider	0.567
	I expect consultation before taking	0.564
	I want to be part of the decision	0.436
	One should only take if prescribed	0.547
F3: Non-prescribed use	Given antibiotics by someone else	0.637
	Community commonly uses without prescription	0.527
	Taken antibiotics without consultation	0.748
	Kept leftover antibiotics	0.784
	Used antibiotics prescribed for someone else	0.774
	Shared antibiotics with others	0.532
	Stop treatment when feeling better	0.673
F4: Understanding of antimicrobial resistance	Too many antibiotics = germs not killed	0.878
	Bacteria become stronger	0.742
	Body gets used to antibiotics	0.910
F5: Attitudes and beliefs	Allowed to demand antibiotics	0.757
	Expect antibiotics to heal quickly	0.715
	Want provider to give antibiotics	0.695

NB: Only loadings ≥0.40 shown. Items loaded strongly onto their intended factors, with minimal cross-loadings, supporting the stability of the exploratory structure.

**Table 5:** Test–retest reliability of CAMUS domains (*n* = 69).

CAMUS Domain	ICC (Two-Way Mixed, Absolute Agreement)	Reliability Interpretation
Understanding of antibiotics	0.85	Excellent
Social and behavioural norms	0.78	Good
Non-prescribed use	0.82	Excellent
Understanding of antimicrobial resistance	0.89	Excellent
Attitudes and beliefs towards antibiotics	0.72	Good

**Table 6:** CFA constructs validity, reliability, and measurement error (refined five-factor model).

Factor	Construct Name	Items	Cronbach’s α	CR	AVE	SD	SEM	SDC	Precision Status
F1	Understanding of antibiotics	7	0.806	0.8	0.36	4.77	2.1	5.82	Acceptable
F2	Social and behavioural norms	8	0.732	0.54	0.18	4.72	2.44	6.78	Low
F3	Non-prescribed use	5	0.808	0.79	0.43	3.76	1.65	4.57	Acceptable
F4	Understanding of AMR	2	0.86	0.85	0.74	1.87	0.7	1.94	Acceptable
F5	Attitudes and beliefs	2	0.619	0.6	0.44	1.7	1.05	2.91	Low
Avg.	Mean across factors	-	-	-	-	-	1.59	4.4	-

NB: CR, composite reliability; AVE, average variance extracted; SD, standard deviation; SEM, standard error of measurement; and SDC, smallest detectable change. Precision is considered acceptable if SEM ≤ SD/2.

**Table 7:** Implications of the CAMUS validation for policy, practice, and future research.

Implication Area	Key Finding	Actionable Recommendation
Policy and surveillance	CAMUS captures behavioural and social drivers (e.g., misuse, community norms, AMR knowledge) that are not included in routine AMR surveillance.	Incorporate CAMUS domains into AMR surveillance frameworks to monitor community-level behavioural drivers over time.
Patient-centred AMS interventions	The five-factor structure shows multiple behavioural determinants (e.g., misuse, social and behavioural norms, expectations).	Design multi-component interventions targeting specific problem domains such as non-prescribed use (F3) or AMR knowledge gaps (F4).
Community engagement	CAMUS was feasible and acceptable (10 min completion).	Use a refined CAMUS version in PHC clinics to gather community data and co-design AMS interventions.
Provider–patient communication	Expectations and trust influence antibiotic-seeking behaviour.	Train clinicians on expectation management, shared decision-making, and literacy-sensitive communication.
Monitoring and evaluation	CAMUS provides structured behavioural indicators suitable for group-level comparisons.	Use CAMUS as a standard outcome measure for AMS programmes targeting patient behaviour.
Future instrument development	Weaker reliability and AVE values in social/attitudinal domains (F2 and F5).	Refine and expand items in these domains; conduct CAMUS 2.0 validation with revised items.

NB: AMR, antimicrobial resistance; AMS, antimicrobial stewardship; AVE, average variance extracted; CAMUS, Community Antimicrobial Use Scale; and PHC, primary healthcare.

**Table 8:** Summary of validation findings and implications for next steps.

Validation Finding	Implication	Required Next Step
Five-factor structure aligned with theory; good reliability for knowledge and misuse domains; moderate reliability for social/attitudinal domains.	CAMUS is suitable for group-level assessment of key behavioural domains.	Retain core structure while refining items in social norms and attitude domains (F2, F5).
AVE < 0.50 for broader behavioural factors; excellent AVE for AMR knowledge.	Broader constructs are conceptually heterogeneous but still coherent; AMR knowledge is tightly defined.	Conduct item-level analysis to strengthen convergent validity, especially in F2, F3, and F5.
Measurement error (SEM/SDC) supports group-, not individual-level, interpretation.	Tool is best suited to surveillance, programme evaluation, and research, not individual diagnosis.	Use CAMUS primarily in epidemiological and programme contexts; refine if individual-level use is needed.
Validation conducted in English and limited geographic settings.	Generalisability across languages and regions is not yet established.	Translate, culturally adapt, and re-validate CAMUS in major South African languages and other LMICs.
Strong association with health literacy and antibiotic-use intentions.	Supports construct validity and relevance for patient-centred stewardship.	Incorporate CAMUS into studies evaluating health literacy and communication interventions.

NB: AMR, antimicrobial resistance; AVE, average variance extracted; CAMUS, Community Antimicrobial Use Scale; F2, social and behavioural norms; F3, non-prescribed use; F5, attitudes and beliefs; LMICs, low- and middle-income countries; SDC, smallest detectable change; and SEM, standard error of measurement.

**Table 9:** Future applications and next phases of CAMUS following validation.

Phase	Focus Area	Rationale	Next Step
Post-validation uses in South Africa	Language and cultural adaptation	The initial validation was appropriately conducted in English, consistent with standard scale-development methodology. Wider implementation requires linguistic and cultural equivalence.	Translate CAMUS into the major South African languages in addition to English, e.g., isiZulu, isiXhosa, and Sepedi, followed by formal cross-cultural adaptation and psychometric re-validation.
Post-validation use in South Africa	Policy and service implementation	CAMUS captures key behavioural drivers of AMR at community level.	Use the refined tool to identify dominant behavioural drivers, e.g., poor knowledge or social and behavioural norms, and tailor pertinent antimicrobial stewardship interventions in PHC settings.
Wider application	Use in other LMICs	Behavioural drivers of antimicrobial use show commonalities across LMIC settings.	Adapt and validate CAMUS for use in other LMICs, particularly in sub-Saharan Africa, with contextual modification and local validation.

NB: CAMUS, Community Antimicrobial Use Scale; AMR, antimicrobial resistance; LMICs, low- and middle-income countries; and PHC, primary healthcare.

**Table 10:** Domains of the 30-Item CAMUS Instrument.

Domain	Description	No. of Items	Response Format
Knowledge	Assesses understanding of appropriate antibiotic use, indications (bacterial vs. viral), side effects, and AMR.	10	Yes/No/Don’t Know
Attitudes and Beliefs	Explores trust in healthcare providers and beliefs about antibiotic necessity.	3	7-point Likert scale *
Motivations	Examines perceived rights or drivers for seeking antibiotics.	2	7-point Likert scale *
Expectations	Probes patient expectations regarding consultation outcomes, provider communication, and symptom relief.	5	7-point Likert scale *
Behavioural Practices	Assesses self-reported behaviours such as adherence, self-medication, and sharing antibiotics.	10	7-point Likert scale *

* Likert scale with response options ranging from ‘Strongly Agree’ to ‘Strongly Disagree’.

## Data Availability

The data that support the findings of this study are not publicly available due to ethical restrictions. However, they are available from the corresponding authors upon reasonable request following completion of the primary analysis and necessary institutional approvals.

## Supplementary Materials

The following supporting information can be downloaded at https://www.mdpi.com/article/10.3390/antibiotics15010107/s1, Supplementary Table S1: Details continued concerns with the prescribing of antibiotics in primary care in South Africa (Refs. [34,35,37,39,40,42,63,64,65,66,67]). Supplementary Box S1: Derivation of measurement error metrics (Refs: [68,69]). Supplementary Table S2: Blank Patient Consent Form.
