# Supplementary material for: Psychometric Validation of the Community Antimicrobial Use Scale (CAMUS) in Primary Healthcare and the Implications for Future Use
Source: Antibiotics (Basel). 2026 Jan 21;15(1):107. doi: 10.3390/antibiotics15010107 (PMC12837195; doi:10.3390/antibiotics15010107)
Supplement: Supplementary file 1 [file antibiotics-15-00107-s001.zip › antibiotics-4063632-supplementary.pdf]

Supplementary Tables

Supplementary Table S1 – Ongoing concerns with the prescribing of antibiotics in primary care in South Africa

Author and year	Aim, objectives and methods	Key results
Farley et al., 2018 [42]	• The objective was to research attitudes, knowledge and practices regarding antibiotics and antibiotic resistance among primary care prescribers<br>• A cross-sectional survey design was used with a self-administered questionnaire<br>• 264 prescribers completed the survey. The vast majority were physicians (98.3%) and practising in the private sector (84.8% of the total)	• 95.8% of prescribers that were interviewed believed antibiotic resistance is a major problem in South Africa<br>• 87.5% expressed a desire for additional education regarding the appropriate use of antibiotics in primary care, with 96.2% also seeking data on local antibiotic resistance patterns to improve future prescribing<br>• Prescribers were also interested in the provision of updated STGs in various formats to improve future prescribing<br>• However, 66.5% of interviewed prescribers felt pressure from their patients to prescribe antibiotics for their infectious disease irrespective whether antibiotics were needed, e.g. for self-limiting viral infections
Gasson et al., 2018 [34]	• The objective was to assess current antibiotic prescribing habits among prescribers in PHCs and compare this against national STGs<br>• A retrospective review of antibiotic prescribing habits was undertaken alongside assessing potential reasons for non-adherence<br>• 654 patient records were reviewed	• Overall, there was appreciable prescribing of antibiotics among patients attending these PHCs - 68.7% of attending patients prescribed an antibiotic<br>• Alongside this, currently low adherence to STGs - adherence only occured in 45.1% of prescriptions<br>• Principal reasons for non-adherence to STGs included : undocumented diagnoses - 30.5% of prescriptions, antibiotics not required including for self-limiting viral infections - 21.6%, incorrect doses prescribed - 12.9%, incorrect duration of antibiotic therapy prescribed - 9.5%, and incorrect antibiotic treatment for the presenting infectious disease - 1.5%
Truter and Knoesen, 2018 [63]	• The aim was to assess primary care antibiotic prescribing habits using a self-administered questionnaire<br>• 16 community pharmacists participated in the study	• Overall, 81.3% of surveyed community pharmacists believed physicians were over-prescribing antibiotic - including for viral infections, potentially enhanced by patient pressure<br>• Amoxicillin /co-amoxiclav were the most prescribed antibiotics - followed by clarithromycin, ciprofloxacin and azithromycin<br>• Surveyed community pharmacists believed sinusitis and URTIs were the most common infectious diseases where antibiotics were being inappropriately prescribed
Lagarde and Blaauw, 2019 [64]	• The objective was to assess prescribing practices for young and healthy simulated patients visiting PHCs with viral bronchitis including both private and public PHCs	• Antibiotics were recommended in 72.6% of consultations, higher in the public sector (78.4%) vs. private sector (66.7%) - exacerbated by perceived patient pressure<br>• This appreciable antibiotic prescribing was despite 84% of prescribers knowing the patient in front of

Author and year	Aim, objectives and methods	Key results
	• Alongsiden this, 125 prescribers were also interviewed using a structured questionnaire across both sectors	them was likely to have a viral rather than bacterial infection (88% in the private sector vs. 77% in the public sector). In addition, 58% knowing that antibiotics would not hasten recovery in patients with viral infections (40% public vs. 68% private; p=0.002)<br>• 47% of prescribers in public PHCs thought patients would not come back if no antibiotic was prescribed – higher in the private sector at 72% (p=0.008) - despite no patient demanding these<br>• Encouragingly, antibiotic prescribing rates among professionals were lower in both sectors (20% lower) in a previous study when healtcare professionals were explicitly told by their patients that they did not want antibiotics for their infection unless they were really necessary – providing encouragement
van Hecke et al., 2019 [65]	• The aim of the study was to determine the perceptions of physicians working in PHCs concerning their prescribing of antibiotics for patients presenting with acute coughs and UTIs alongside their experiences concerning point-of-care testing to aid diagnosis<br>• Qualitative interviews were undertaken among 23 prescribers	• Antibiotic prescribing decisions were typically influenced by a number of factors. These included (1) their clinical assessment of presenting patients, (2) patients' comorbidities and (3) their perceptions regarding the expectations of their patients<br>• There were observed difficulties in the communication between prescribers and patients – this often hampered efforts by physicians to explain non-antibiotic management strategies to patients including for self-limiting viral infections<br>• In view of these findings, participating physicians were typically positive towards current and future point-of-care testing, especially for viral infections, to improve evidence-based antibiotic prescribing<br>• However, there were concerns with current resources and workflow issues – which would influence any subsequent uptake and use of point-of-care tests as part of their routine care of patients presenting to PHCs with infectious diseases
Balliram et al., 2021 [66]	• The objective was to assess the knowledge, attitudes and practices of doctors, pharmacists and nurses regarding the use of antimicrobials, antimicrobial resistance and antimicrobial stewardship practices<br>• A national online survey was conducted among doctors, pharmacists and nurses	• Overall, 96.4% of doctors taking part in the survey believed AMR was a severe global threat, with 96.6% also believing AMR is a significant problem in South Africa<br>• However, only 37.70% of participating doctors felt ≤ 50% confidence in their knowledge regarding antimicrobials, AMR and AMS<br>• Having said this, encouragingly 94.9% of doctors believed antibiotics were not effective against viral infections (vs. e.g. 75.3% for nurses) and 99.1% that common colds are caused by viruses (vs. e.g. 90.2% nurses).<br>• 91.61% of participating professionals believed the overuse of antimicrobials was the greatest

Author and year	Aim, objectives and methods	Key results
		contributor to AMR, followed by patient pressure (75.26%) and non-adherence to prescribed treatments (73.26%)<br>• Overall, 80.1% of participating professionals articulated a need for more education and training on antimicrobial use, AMR, and AMS – especially as 91.22% of participating doctors saw educational campaigns, 84.72% STGs, and 66.31% improved infection control measures, as important strategies to combat AMR.
Govender et al., 2021 [67]	• The objective was to evaluate the use and implementation of the STGs/EML) among prescribers (nurses) at a public tertiary institution and associated PHC facilities<br>• A mixed approach was used- including evaluating patient records and undertaking interviews using a structured questionnaire	• 41% of surveyed nurses had access to the latest STG/EML – with all the participating nurses stated they often/ sometimes refer to available STGs/EML when managing patients with infectious diseases. However, only 41% stated they had access to the latest STG/EML<br>• There was a 59.7% adherence rate for prescriptions to the STG/EML.<br>• Having said this, 94.9% of surveyed nurses requested training on the use of STGs/EML to improve their future prescribing. This included antibiotics/ infectious diseases as most of those surveyed had not received any formal training on its use
Alabi et al, 2022 [37]	• The aim was to assess the appropriateness of antibiotic prescribing among practicing GPs in the private sector<br>• This included an analysis of antibiotic prescriptions (188,141) in the notes of 174,889 patients<br>• Appropriateness based on the ICD-10 classification and whether an antibiotic was deemed warranted or not	• 92.9% of assessed patients were prescribed one antibiotic by their primary care GPs when attending their PHC, with 7.1 % prescribed two or more antibiotics<br>• Penicillins were the most prescribed antibiotics (40.7% of all antibiotics prescribed), followed by macrolides (16.8%) and cephalosporins (15.7% - all generations combined)<br>• 46.1% of all diagnoses made included diseases of the respiratory system<br>• Only 8.8% of all antibiotic prescriptions were subsequently deemed as appropriate and 32.0% potentially appropriate<br>• Overall, 45.4% of antibiotic prescriptions were deemed inappropriate and 13.8% could not be assessed due to a lack of specific codes/ contained unlisted codes/ or contained unclear descriptions in the prescription
Guma et al., 2022 [35]	• The aim was to assess current antibiotic empiric prescribing habits among private GPs among patients attending with ARIs - and associated key factors<br>• A semi-structured web-based questionnaire was used	• 209 GPs took part in the survey<br>• 55.5% of surveyed GPs admitted to prescribing antibiotics empirically for patients with ARIs more than 70% of the time - primarily for symptom relief and the prevention of complications<br>• Encouragingly, GPs with more experience and working alone were slightly less likely to prescribe antibiotics empirically

Author and year	Aim, objectives and methods	Key results
		• Key factors that were significantly associated with empiric antibiotic prescribing were diagnostic uncertainty, workload/time pressures and the use of a formulary
Chigome et al, 2025 [39]	• A point prevalence survey was undertaken among PHCs in two Provinces in South Africa and repeated<br>• This was part of a larger study	• Data for 615 patients were recorded and evaluated<br>• The most common symptoms where antibiotics were prescribed included a genital discharge (21.8%), painful urination (18.4%), acute cough (17.7%), and a sore throat (13.5%). Patients could potentially have more than one symptom<br>• At least one antibiotic was prescribed for 87.0% of attending patients, with Access antibiotics accounting for 53.4% of antibiotics prescribed. 46.6% were from the Watch group<br>• Ceftriaxone (29.7%), amoxicillin (29.4%) and azithromycin (28.4%) were the most frequently prescribed antibiotics among presenting patients
Maluleke et al, 2025 [40]	• The objective was to assess antibiotic dispensing practices in a rural Province<br>• 75.7% (128/169) of operational pharmacies took part in this questionnaire-based study<br>• 78.3% response rate – with 106 pharmacists and 207 pharmacist assistants taking part	• Antibiotics accounted for 47.9% of all medicines dispensed, with penicillins the most dispensed antibiotic (41.1%)<br>• 47.2% of antibiotics dispensed included cephalosporins, macrolides and fluoroquinolones – typically Watch antibiotics, with STIs (33.5%) and URTIs (25.8%) the most frequent believed indication for antibiotics<br>• Encouragingly, 98.1% of community pharmacists and 97.6% of pharmacist assistants indicated they always or mostly offered symptomatic relief before suggesting/ dispensing antibiotics without a prescription to patients with typically self-limiting conditions such as URTIs

NB: ABR = Antibiotic Resistance; AMR = Antimicrobial Resistance; AMS = Antimicrobial Stewardship; ARI = Acute Respiratory Infection; AWaRe = Access, Watch and Reserve [17]; EML = Essential Medicines List; GPs = General Practitioners; PHCs = Primary Healthcare Clinics; RTIs = Respiratory Tract Infections; SPs = Simulated Patients; STGs = Standard Treatment guidelines; STIs = Sexually Transmitted Infections; URTIs = Upper Respiratory Tract Infections; UTIs = Urinary Tract Infection

Supplementary Box 1: Derivation of measurement error metrics

To evaluate the interpretability of the CAMUS instrument, we calculated the Standard Error of Measurement (SEM) and the Smallest Detectable Change (SDC) for each factor using the standard deviation (SD) and reliability coefficients (Cronbach's alpha) obtained from the study sample (N=1283).[68,69]

1. Standard Error of Measurement (SEM)

The SEM quantifies the precision of the individual scores and was calculated using the formula:

SEM = SD.$\sqrt{(1-\alpha)}$

Where:

- **SD** is the standard deviation of the specific factor.
- **α** is Cronbach's alpha (internal consistency reliability) for that factor.

2. Smallest Detectable Change (SDC)

The SDC (also referred to as the Minimal Detectable Change, MDC) indicates the smallest change in score that can be considered real and beyond measurement error (at the 95% confidence level). It was calculated as:

SDC = 1.96 . $\sqrt{2}$. SEM

Where:

- **1.96** represents the z-score for the 95% confidence interval.
- **√2** = square root of 2

Supplementary Table S2 Consent form

You are hereby kindly invited to participate in a Research Project

Name of Project: **Development and validation of a Community Antimicrobial Use Scale (CAMUS) for use at primary health care level in South Africa**

I have been informed about the aims and objectives of the proposed study on the development and validation of a scale for antimicrobial use within the community at primary healthcare level in South Africa. I have had the opportunity to ask any questions and have been given adequate time to consider whether or not to participate. I have not been coerced or pressured into taking part in any way.

I understand that my participation in this study may involve sound recordings being taken of me, and that this material may be used in scientific publications that will be electronically available worldwide. I consent to this, provided that my name is not revealed.

I understand that my participation in this project is completely voluntary, and I may withdraw from it at any time without giving any reason. This will not affect the regular treatment that I receive for my condition, nor will it influence the care that I receive from my regular doctor.

I am aware that this Project has received approval from the Sefako Makgatho University Research Ethics Committee (SMUREC), Sefako Makgatho Health Sciences University. I understand that the results of this project will be used for scientific purposes and may be published. I agree to this, provided that my privacy is protected.

I hereby give my consent to participate in this Project.

....

Name of patient/volunteer Signature of participant

....

Place Date Witness

Statement by the Researcher

I have supplied both verbal and written information pertaining to this Project.

I hereby consent to answer any forthcoming questions to the best of my ability.

Furthermore, I commit to following the approved protocol.

Ms N Ramdas ....

Name of Researcher Signature Date Place
